# MiR-RACE, a New Efficient Approach to Determine the Precise Sequences of Computationally Identified Trifoliate Orange (*Poncirus trifoliata*) MicroRNAs

**DOI:** 10.1371/journal.pone.0010861

**Published:** 2010-06-07

**Authors:** Changnian Song, Jinggui Fang, Chen Wang, Lei Guo, Kibet Korir Nicholas, Zhengqiang Ma

**Affiliations:** 1 College of Horticulture, Nanjing Agricultural University, Nanjing, China; 2 State Key Laboratory of Crop Genetics and Germplasm Enhancement, Nanjing Agricultural University, Nanjing, China; East Carolina University, United States of America

## Abstract

**Background:**

Among the hundreds of genes encoding miRNAs in plants reported, much more were predicted by numerous computational methods. However, unlike protein-coding genes defined by start and stop codons, the ends of miRNA molecules do not have characteristics that can be used to define the mature miRNAs exactly, which made computational miRNA prediction methods often cannot predict the accurate location of the mature miRNA in a precursor with nucleotide-level precision. To our knowledge, there haven't been reports about comprehensive strategies determining the precise sequences, especially two termini, of these miRNAs.

**Methods:**

In this study, we report an efficient method to determine the precise sequences of computationally predicted microRNAs (miRNAs) that combines miRNA-enriched library preparation, two specific 5′ and 3′ miRNA RACE (miR-RACE) PCR reactions, and sequence-directed cloning, in which the most challenging step is the two specific gene specific primers designed for the two RACE reactions. miRNA-mediated mRNA cleavage by RLM-5′ RACE and sequencing were carried out to validate the miRNAs detected. Real-time PCR was used to analyze the expression of each miRNA.

**Results:**

The efficiency of this newly developed method was validated using nine trifoliate orange (*Poncirus trifoliata*) miRNAs predicted computationally. The miRNAs computationally identified were validated by miR-RACE and sequencing. Quantitative analysis showed that they have variable expression. Eight target genes have been experimentally verified by detection of the miRNA-mediated mRNA cleavage in *Poncirus trifoliate*.

**Conclusion:**

The efficient and powerful approach developed herein can be successfully used to validate the sequences of miRNAs, especially the termini, which depict the complete miRNA sequence in the computationally predicted precursor.

## Introduction

One of the most important developments in molecular biology over the past two decades is the emerging picture of a new layer of gene regulation under the control of small yet versatile RNAs [1]. Small RNA (sRNA) molecules are widely recognized as common and effective modulators of gene expression in many eukaryotic organisms. According to the current knowledge, sRNAs are generally divided into several categories, including microRNAs (miRNAs), short interfering RNAs (siRNAs), trans-acting siRNAs (ta-siRNAs), natural antisense transcript siRNAs (nat-siRNAs), and Piwi-interacting RNAs (piRNAs) in metazoans [2]. In plants, microRNAs (miRNAs) are produced from partially complementary dsRNA precursor molecules [3], [4]. These plant miRNAs are the best-characterized sRNAs, and the pathways by which they are generated and their roles in gene regulation have been well documented [2], [3], [5]. Several hundred genes encoding miRNAs in plants have been experimentally identified by the traditional Sanger sequencing method, and increasingly more are predicted by numerous computational methods. These methods mainly use secondary structural information to search expressed sequence tags (ESTs) and to mine the repository of available genomic sequences [6]–[11], and they have obvious advantages, including the quick prediction of a large number of miRNAs, low costs, and the prediction of novel and non-abundant miRNAs that are usually difficult to clone directly. However, the miRNA prediction algorithms often cannot predict the accurate location of the mature miRNA in a precursor with nucleotide-level precision. Even though false-positive predictions have been minimized using various scores and rank cutoffs, the precise sequences usually cannot be determined and several candidate miRNA orthologs or paralogues might be predicted for a specific miRNA. Unlike protein-coding genes defined by start and stop codons, the ends of miRNA molecules do not have characteristics that can be used to define the mature miRNAs exactly. The determination of the precise sequence of the mature miRNA, including the ends, is essential for downstream research applications in various organisms, such as miRNA target prediction and further studies on miRNA evolution, the regulatory role of miRNAs, and the mechanism of miRNA biogenesis. Mutations in the seed region of human miR-96 have a strong impact on miR96 biogenesis and result in a significant reduction in miRNA targeting [12], confirming that it is important to determine the precise sequence of mature miRNAs before using them in further studies.

In previous studies, a combination of computational prediction and experimental verification was used to identify miRNAs, in which the experimental validation was mainly focused on determining the expression of the miRNAs by the robust techniques of RNA blotting and/or RT-PCR. Notably, these two techniques can only confirm the existence and size, but not the full precise sequence, of a miRNA predicted computationally. With a greater number of new potential miRNAs predicted by bioinformatics approaches and deposited in the miRBase Sequence Database (http://microrna.sanger.ac.uk/sequences/), the precise sequences of the homologs and/or orthologs of the miRNAs cloned from model organisms need to be determined before the initiation of further studies on their functions and biogenesis. To our knowledge, no reports have employed a comprehensive strategy to determine the precise sequences of the miRNAs computationally predicted. We developed an integrative approach combining the strategies of a miRNA-enriched library preparation, 5′ RACE and 3′ RACE reactions, and sequence-directed cloning, which made it possible to determine the sequences of even the non-abundant miRNAs that are typically difficult to clone directly. This is the first report of the validation of the up- and downstream nucleotides flanking the last candidate nucleotide in the predicted miRNA.

**Figure 1:**
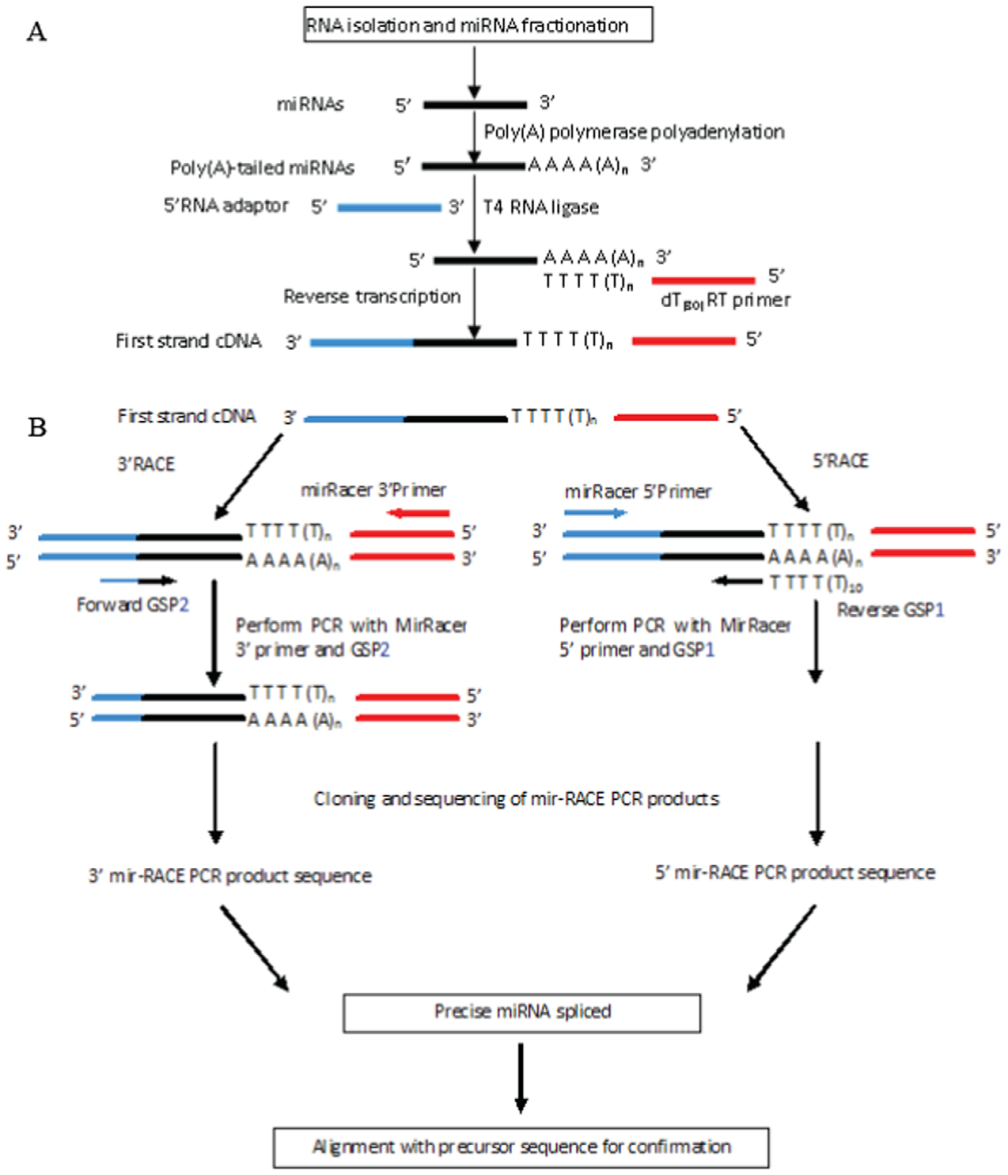
a. Determination of the precise miRNA sequence by 5′ and 3′ miR-RACE. (a) miRNA cDNA library construction. (b) Analysis of miRNA 5′RACE and 3′RACE. Sequences for the 5′RNA adaptor, dT(30)RT primer, MirRacer 3′ Primer, MirRacer 5′ Primer, GSP1, and GSP2 are listed in Table 2.

The method we developed comprises the following main steps: (i) miRNA-enriched library preparation; (ii) 5′ miR-RACE and 3′ miR-RACE for accurate amplification of the 5′ and 3′ ends of a miRNA; (iii) PCR product cloning and sequencing; (iv) real-time PCR (RT-PCR) of the miRNAs using the primers derived from the validated miRNA sequences; (v) cloning and sequencing of the RT-PCR products for the validation of the PCR products. The schematic flowchart of this strategy for precise miRNA sequence determination is shown in Fig. 1. The innovative core steps in our method are the two PCR reactions amplifying the 5′ and 3′ ends of the miRNA, respectively, in which two specific primers cover both parts of the candidate miRNA and adaptor. These two PCR reactions are denoted as 5′- and 3′-miR-RACE based on their similarity to the rapid amplification of cDNA ends (RACE) technique. The efficiency of this method was validated well using nine trifoliate orange, one of the most important rootstocks of citrus, miRNAs (ptr-miRNAs) that were predicted computationally.

## Results

### Prediction of potential ptr-miRNAs

**Figure 2:**
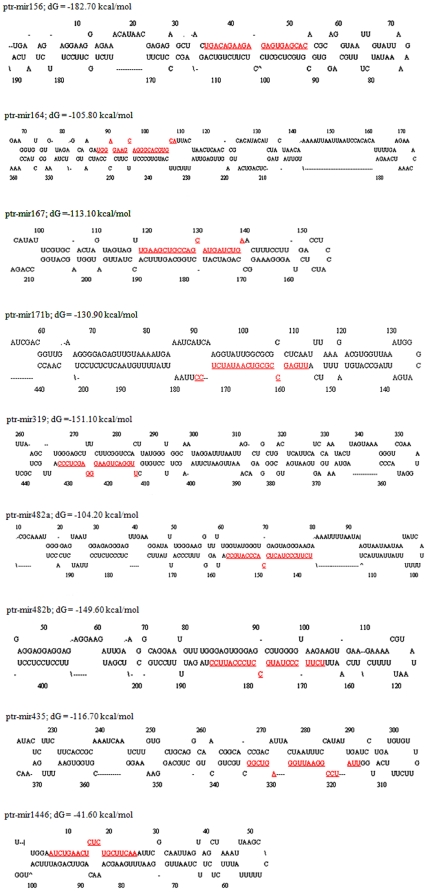
Predicted fold-back structures of the identified ptr-miRNAs. The mature miRNA sequences are shaded. The miRNA precursors may be slightly longer than the sequences shown in this figure. Predicted fold-back structures of the identified ptr-miRNAs. The mature miRNA sequences are shaded. The miRNA precursors may be slightly longer than the sequences shown in this figure.

The method used in the computational identification of ptr-miRNAs was similar with the methods used by Zhang *et al.* [6] and Sunkar *et al.* [13], by which nine potential ptr-miRNAs (Table 1) were predicted. Subsequently, we re-checked these candidate ptr-miRNAs manually following our work on citrus [14], and the predicted precursor secondary structures of these ptr-miRNAs, as an important validation parameter for MIR genes, are presented in Fig. 2.

**Table 1:** Summarized results of the validation of the *P. trifoliate* miRNA sequences.

Pt-miRNAs	Sequence in PT	miRNAs in Arabidopsis or other plants	Computational prediction	Region of precursor	Sequencing replicates	5′-RACE nt (frequency)	
						PT	Arabidopsis
ptrmir156	UGACAGAAGAGAGUGAGCAC	UGACAGAAGAGAGUGAGCAC	UGACAGAAGAGAGUGAGCAC	UACUGACAGAAGAGAGUGAGCACACG	3	1(1/16); 10(14/16)	10(8/12);9(4/12)
ptrmir164	UGGAGAAGCAGGGCACGUGCA	UGGAGAAGCAGGGCACGUGCA	UGGAGAAGCAGGGCACGUGCA	GAUGGAGAAGCAGGGCACGUGCAUU	3	9(2/16); 10(12/16); 12(2/16)	10(13/13)
ptrmir167	UGAAGCUGCCAGCAUGAUCUGA	UGAAGCUGCCAGCAUGAUCUGA	UGAAGCUGCCAGCAUGAUCUGA	GUUGAAGCUGCCAGCAUGAUCUGAA	3	9(12/15)	9(9/12)
ptr-mir171	UUGAGCCGCGUCAAUAUCUCC	UUGAGCCGUGCCAAUAUCACG	UUGAGCCGCGUCAAUAUCUCC	UCAUUGAGCCGCGUCAAUAUCUCCUU	3	13(10/20);10(10/20)	12(1/12); 11(1/12); 10(10/12)
			UGAGCCGCGUCAAUAUCUCCU		3		
ptrmir319	UUGGACUGAAGGGAGCUCCC	UUGGACUGAAGGGAGCUCCC	UUGGACUGAAGGGAGCUCCC	UGCUUGGACUGAAGGGAGCUCCCAUU	3	10(15/15)	10(13/13)
ptrmir482a	UCUUCCCUACUCCACCCAUGCC	UCUUCCCUACUCCUCCCAUUCC	UCUUCCCUACUCCACCCAUG	AUAUCUUCCCUACUCCACCCAUGCCAU	3	10(17/18)	8(7/8)
			UCUUCCCUACUCCACCCAUGCC		3		
ptrmir482b	UCUUCCCUAUGCCUCCCAUUCC	UCUUCCCUACUCCUCCCAUUCC	UCUUCCCUAUGCCUCCCAUUCC	AAUUUCUUCCCUAUGCCUCCCAUUCCUAU	3		8(8/9)
			UCUUCCCUAUGCCUCCCAUU		3		
ptrmir1446	AUCUGAACUCUCUGCUUCAA	UUCUGAACUCUCUCCCUCAA	AUCUGAACUCUCUGCUUCAA	UGGAAUCUGAACUCUCUGCUUCAAAUU	3	10(12/16)	13(10/10)
ptrmir435	UUAUCCGGAAUUGGAGUCGG	UUAUCCGGUAUUGGAGUUGA	UUAUCCGGAAUUGGAGUCGG	GGUUAUCCGGAAUUGGAGUCGGCUG	3		

### 5′ miR-RACE and 3′ miR-RACE PCR products of ptr-miRNAs

To amplify the 5′ and 3′ ends of the target miRNA, procedures similar to the rapid amplification of cDNA ends (RACE) were employed (Fig. 1B). The difference between our method and traditional RACE lies in the gene-specific primers used (Table 2). The two gene-specific primers were designed considering two additional parameters. The first parameter was that the primers covered 17 nucleotides of the candidate mature miRNAs, and these 17 nucleotides were specific to the corresponding miRNA and met the criterion for the minimum number of nucleotides of a regular PCR primer. The second parameter was that mismatches between the sequence of the specific primer and that of the end sequence of the real miRNAs were allowed, and that these mismatches should not influence the PCR amplification, similar to the principle employed in site-directed mutagenesis [15], [16] and in the addition of restriction sites to the termini of amplified DNA employed in recombinant DNA technology [17]. Furthermore, the workability of the miR-5′ RACE and miR-3′ RACE reactions was validated by the application of primers designed to have 1–3 nucleotides mismatched to the end sequence of the real miRNAs (Supplementary Table S1; Supplementary Fig. S1). This suggested the PCR reactions could allow the application of primers that might have 1–3 nucleotides mismatches to the precise sequence of the real corresponding miRNAs to be verified. We chose to use 17 rather than all of the nucleotides of the miRNA for primer design based on the hypothesis that four or fewer nucleotides flanking the last nucleotide in the predicted miRNA would vary, thus maintaining at least 75% identity between the primer and the miRNA orthologs, consistent with the conservation reported for cloned miRNA homologs. This design would allow at most three and four mismatched nucleotides and, if found to be true, should be validated.

**Table 2:** Primers used for miR-5′ RACE, miR-3′ RACE, and real-time PCR.

Pt-miRNAs	GSP1 (5′→3′)	GSP2 (5′→3′)	GSP3 (5′→3′)
ptrmir156	TTTTTTTTTTGTGCTCACTCTCTTCTG	GGAGTAGAAATGACAGAAGAGAGTGAG	TGACAGAAGAGAGTGAGCAC
ptrmir164	TTTTTTTTTTGCACGTGCCCTGCTTCT	GGAGTAGAAATGGAGAAGCAGGGCACG	TGGAGAAGCAGGGCACGTGCA
ptrmir167	TTTTTTTTTTTCAGATCATGCTGGCAG	GGAGTAGAAATGAAGCTGCCAGCATGA	TGAAGCTGCCAGCATGATCTGA
ptr-mir171	TTTTTTTTTTGGAGATATTGACGCGGC	GGAGTAGAAATTGAGCCGCGTCAATAT	TTGAGCCGCGTCAATATCTCC
ptrmir319	TTTTTTTTTTGGGAGCTCCCTTCAGTC	GGAGTAGAAATTGGACTGAAGGGAGCT	TTGGACTGAAGGGAGCTCCC
ptrmir482a	TTTTTTTTTTCATGGGTGGAGTAGGGA	GGAGTAGAAATCTTCCCTACTCCACCC	TCTTCCCTACTCCACCCATGCC
ptrmir482b	TTTTTTTTTTAATGGGAGGCATAGGGA	GGAGTAGAAATCTTCCCTATGCCTCCC	TCTTCCCTATGCCTCCCATTCC
ptrmir1446	TTTTTTTTTTTTGAAGCAGAGAGTTCA	GGAGTAGAAAATCTGAACTCTCTGCTT	ATCTGAACTCTCTGCTTCAA
ptrmir435	TTTTTTTTTTCCGACTCCAATTCCGGA	GGAGTAGAAATTATCCGGAATTGGAGT	TTATCCGGAATTGGAGTCGG

GSP1 is the specific primer for miR-5′ RACE, and the underlined region base pairs with the 3′ poly(A)n; GSP2 is the specific primer used for miR-3′ RACE, and the underlined region base pairs with the 5′ adaptor; GSP3 is the specific primer used for miRNA quantitative real-time PCR (qRT-PCR).

**Figure 3:**
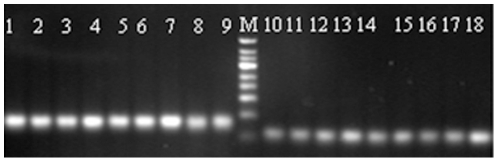
The 3′ RACE and 5′ RACE products of ptr-miRNAs amplified by PCR are shown in an ethidium bromide-stained agarose gel. The sizes of the molecular weight markers of the bottom and the second from bottom bands are 50 bp and 100bp, respectively. Lanes 1–9 are 3′RACE products of ptr-miR156, ptr-miR164, ptr-miR167, ptr-miR171, ptr-miR319, ptr-miR482a, ptr-miR482b, ptr-miR435, and ptr-miR1446, respectively, and lanes 10–18 are the 5′RACE products of them.

The GSP1 and GSP2 also included ten nucleotides of Poly(T) and ten nucleotides of the adaptor sequence, respectively, for longer primers of up to more than 21 nucleotides. These modifications resulted in a high specificity and a better match between the annealing temperatures of the specific primer and the opposite reverse adaptor primer, which were the most technically challenging steps in this miR-RACE. By using one specific primer and one reverse primer during PCR, the precise sequence of the end of the miRNA opposite to the specific primer could be correctly amplified and validated. In this study, the 17 nucleotides complimentary to the miRNA were sufficient for the accurate and efficient PCR amplification of the opposite ends, and the mismatches within the gene specific primer (GSP) did not influence the aim of this work, to PCR-amplify the two ends of the ptr-miRNAs (Fig. 3). The anticipated sizes of the PCR products of the nine ptr-miRNAs were estimated during the prediction of the ptr-miRNAs, and the identity of these PCR products were validated by the subsequent cloning and sequencing.

### Cloning and sequencing of the 5′ miR-RACE and 3′ miR-RACE PCR products

All the miR-RACE PCR products were cloned and sequenced, and all of them yielded reliable sequences. For a relatively high confirmation of the sequences of the cloned products, three clones of each PCR product were sequenced. The sequences of each pair of 5′ miR-RACE and 3′ miR-RACE PCR products of every one of the nine ptr-miRNAs were spliced to generate the whole mature miRNA sequences (Table 3).

**Table 3:** Alignment between ptr-miRNAs and their orthologs in *Arabidopsis*.

miRNA(5′→3′)	Nucleotide order																					
	1	2	3	4	5	6	7	8	9	10	11	12	13	14	15	16	17	18	19	20	21	22
mir156	U	G	A	C	A	G	A	A	G	A	G	A	G	U	G	A	G	C	A	C		
ptr-mir156																						
mir164	U	G	G	A	G	A	A	G	C	A	G	G	G	C	A	C	G	U	G	C	A	
ptr-mir164																						
mir167	U	G	A	A	G	C	U	G	C	C	A	G	C	A	U	G	A	U	C	U	G	A
ptr-mir167																						
mir171	U	U	G	A	G	C	C	G	U	G	C	C	A	A	U	A	U	C	A	C	G	
ptr-mir171b									C		U								U		C	
mir319	U	U	G	G	A	C	U	G	A	A	G	G	G	A	G	C	U	C	C	C		
ptr-mir319																						
mir482	U	C	U	U	C	C	C	U	A	C	U	C	C	U	C	C	C	A	U	U	C	C
ptr-mir482a														A						G		
mir482	U	C	U	U	C	C	C	U	A	C	U	C	C	U	C	C	C	A	U	U	C	C
ptr-mir482b										U	G											
mir1446	U	U	C	U	G	A	A	C	U	C	U	C	U	C	C	C	U	C	A	A		
ptr-mir1446	A													G		U						
mir435	U	U	A	U	C	C	G	G	U	A	U	U	G	G	A	G	U	U	G	A		
ptr-mir435									A									C		G		

The cloning and sequencing of the miR-RACE PCR products were performed according to the instruction for the TOPO TA cloning Kit (Invitrogen, USA). The sequencing results were also used to confirm the predicted ptr-miRNAs and to identify the precise end sequences of them (Table 3). The sequence identity between the cloned and validated miRNA and the region in the corresponding precursor was also used to confirm the success of the 5′ miR- and 3′ miR-RACE technique for the precise determination of the miRNA sequences. The sequencing results demonstrated that the ptr-miRNAs were conserved relative to those of *Arabidopsis* and the Poplar (Table 3), but not all at the 100% identity level. The nine ptr-miRNAs analyzed in this study exhibited more variation at the terminal nucleotides than in the internal nucleotides of the miRNAs. This is in agreement with the results obtained in a study by Seitz *et al.* [18]. A possible explanation for the sequence variation across these small active elements is that transcription and miRNA processing might introduce differences at both ends and in the internal regions of the miRNA. The sequence validation results also demonstrated that the four conserved miRNAs (ptr-mir156, 164, 167, and 319) were identical both in length and nucleotide sequence with their orthologs in *Arabidopsis*, but the other five non-conserved miRNAs varied in sequence at both internal and terminal nucleotides.

### Expression analysis of ptr-miRNAs

**Figure 4:**
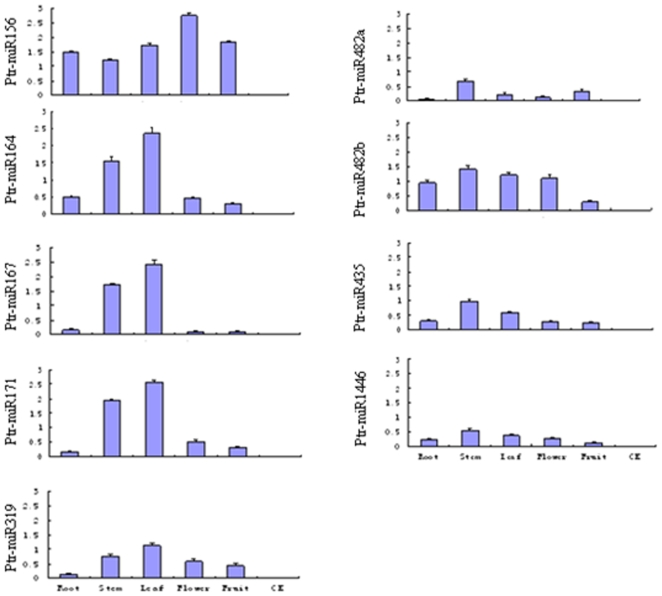
Relative expression levels of trifoliate orange miRNAs in different the trifoliate orange tisues of root, stem, leaf, flower, and fruit. Each reaction was repeated three times and the template amount was corrected by 5.8 s rRNAs.

An expression analysis of the nine trifoliate orange miRNAs was also carried out in this study for a more comprehensive and efficient characterization of the miRNAs, using shared primers and the same miRNA library, which has been one of the most important works to be studied on miRNAs. After the validation of the precise sequence of nine ptr-miRNAs, the primers for the RT-PCR were synthesized (Table 2). The preferential expression of a miRNA in specific tissues might provide clues about its physiological function. All nine ptr-miRNAs exhibited expression patterns (Fig. 4) similar to their orthologs in *Arabidopsis* and Poplar. The majority of the miRNAs were expressed ubiquitously in all tissues, and some were expressed in tissue-, species-, and/or growth-stage-specific patterns. All of the RT-PCR reaction products were cloned and sequenced for positive validation.

### Identification of miRNA-guided cleavage of target mRNAs in trifoliate orange

**Figure 5:**
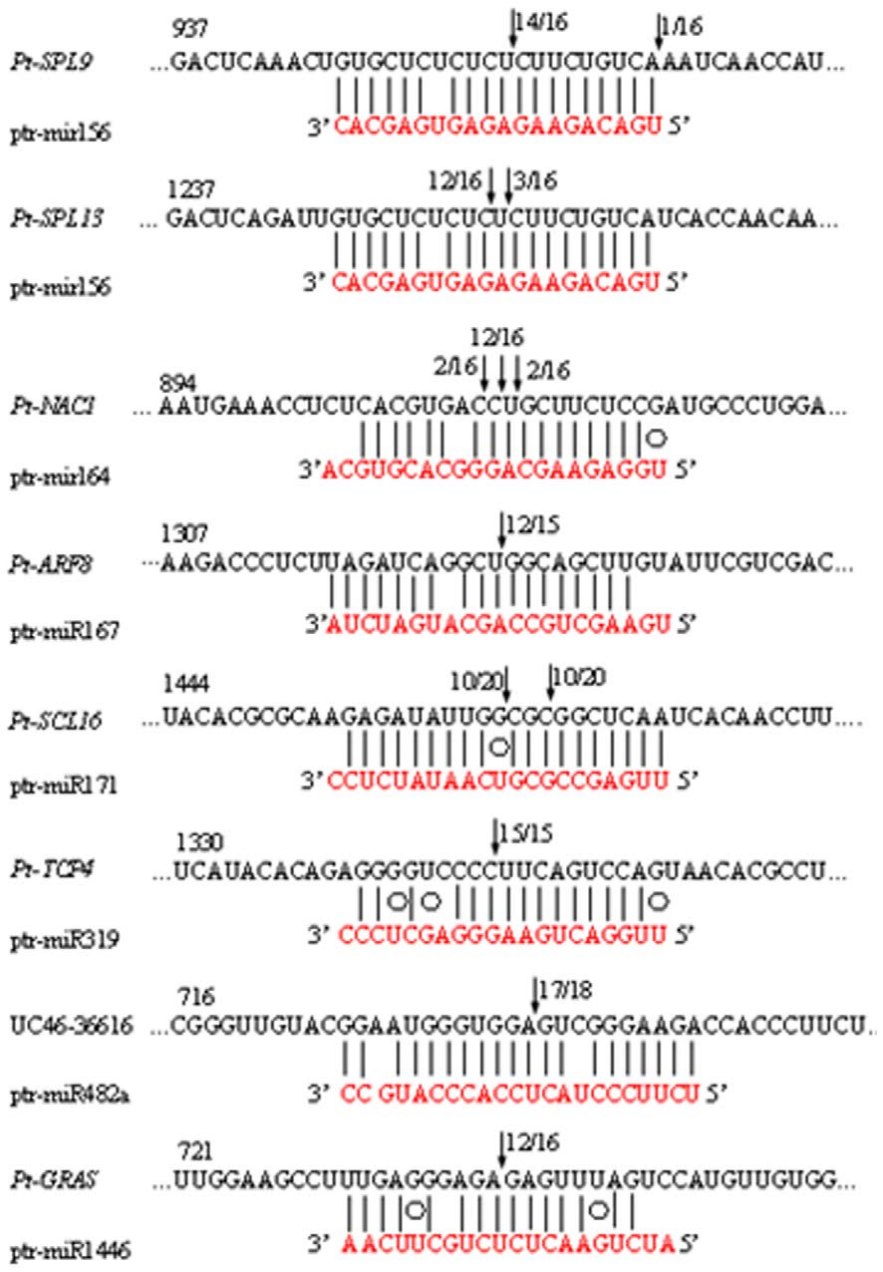
Mapping of the mRNA cleavage sites by RNA ligase-mediated 5′ RACE. Each top strand (black) depicts a miRNA complementary site, and each bottom strand depicts the miRNA (red). Watson-Crick pairing (vertical dashes) and G∶U wobble pairing (circles) are indicated. The arrows indicate the 5′ termini of mRNA fragments isolated from citrus, as identified by cloned 5′RACE products, with the frequency of clones shown. Only the cloned sequences that matched the correct gene and had 5′ ends within a 100 nt window centered on the miRNA validation are included (Table 1). The miRNA sequence shown corre (1 out of 4 PCR clones) is indicated in lower case and corresponds to the most common miRNA supported by the miRNA PCR. RNA ligase-mediated 5′RACE was used to map the cleavage sites. The partial mRNA sequences from the target genes were aligned with the miRNAs. The numbers indicate the fraction of cloned PCR products terminating at different positions. *Pt-SPL9* (accession FJ502237), *Pt-SPL13* (accession FJ502238), *Pt-NAC1* (accession FJ619349), *Pt-ARF8* (UC46-16450), *Pt-SCL6* (accession GQ505957), *Pt-TCP4* (GQ505958), *Pt-GRAS* (accession FC901464). *Pt-SPL9* (accession FJ502237) was similar to AT2G42200 (NM_129782) SPL9 (squamosa promoter-binding protein 9); *Pt-SPL13* (accession FJ502238) was similar to AT5G50670 (NM_124445) SPL (squamosa promoter-binding protein); *Pt-NAC1* (accession FJ619349) was similar to AT5G61430 ( NM_125536) ANAC100 (ARABIDOPSIS NAC DOMAIN CONTAINING PROTEIN 100); *Pt-ARF8* (UC46-16450) was similar to AT5G37020 (NM_001085203) ARF8 (AUXIN RESPONSE FACTOR 8); *Pt-SCL6* (accession GQ505957) was similar to AT4G00150 (NM_116232) SCL6 (scarecrow-like transcription factor 6); *Pt-TCP4* (GQ505958) was similar to AT3G15030 (NM_180258) TCP4 (TCP family transcription factor 4); UC46-36616 was similar to AT1G12220 (NM_101094) RPS5 (RESISTANT TO P. SYRINGAE 5); *Pt-GRAS* (accession GU072592) was similar to IPR005202 (XM_002318667) GRAS71 (GRAS family transcription factor).

The highly exact complementarity between the ends of the miRNAs and their substrate probably allows them to interact with greater specificity to their substrate mRNAs without the need for stronger complementarity throughout the miRNA or a larger overlap. The differences in the miRNA sequences could influence the directed-digestion sites on target mRNAs. If variation is introduced in the terminal nucleotides of miRNAs, it is unclear whether or not their function will also change. Most of the *Arabidopsis* miRNAs have been shown to guide cleavage of their target genes [19]–[21]. To verify the nature of the predicted ptr-miRNA targets and to study how the ptr-miRNAs regulate their target genes, RLM-RACE experiment was employed, which was carried out in this study for better characterization of the ptr-miRNAs predicted. It is also one of the most common and widely used methods in the literatures [22], [23] to support bioinformatics data. All nine of the ptr-miRNAs guided the target cleavage, most often at the tenth nucleotide, as expected (Fig. 5). From the precise sequences of the ptr-miRNAs results, we know that the miRNA-guided cleavage in trifoliate orange obeyed the principle that base-paring to the 5′ ‘seed’ region of the miRNA was the dominant factor for the miRNA target recognition, and that the cleavage site was mostly located at the tenth nucleotide, just 3′ of the ‘seed’ sequence [24]. All the nine predicted targets were found to have specific cleavage sites corresponding to the miRNA complementary sequences and might be regulated by the miRNAs in the style of small interfering RNAs (siRNAs) [25] directing the cleavage of mRNA targets with extensive complementarity to the miRNAs [22].

### Conclusions

Our study introduces a new, efficient strategy to verify the real sequences of mature miRNAs that were predicted computationally. Based on the sequencing results for a test set of miRNAs from trifoliate orange, we further suggest that there could be variation between the sequences of homologous miRNAs from different plant species. Theoretically, it is easy to understand that miRNAs could be changing during evolution across the plant kingdom. The sequences generated via the miR-RACE PCR experiments matched the predicted miRNA sequences located in the corresponding precursors used for stem-loop prediction, and these miRNAs were also complementary to the target sequences with fewer than three mismatches in trifoliate orange (Fig. 5). This suggests that the sequence variation of the miRNAs might not have changed their function, since there may be enough complimentarity remaining for these miRNAs to work on their target gene transcripts. The sequence variation of the miRNAs could also be due to evolutionary factors.

In summary, this method functions as a complementary approach to all of the computational miRNA prediction methods developed for miRNA sequence determination, making it possible to clone an interesting miRNA and study the conservation of that miRNA in the plant kingdom. The results from the ptr-miRNA study suggest that it is wise to validate the precise sequences of computationally predicted miRNAs before initiating further experimental studies on the miRNA. The method we proposed herein is a powerful tool for miRNA sequence, especially miRNA termini, determination. Theoretically, it is better to validate every one miRNA predicted computationally for some further study on its regulation function, for a different nucleotide may influence the function of it.

## Materials and Methods

### miRNAs, ESTs, cDNA, and mRNA sequences

All of the 1894 known plant miRNAs, which have been validated by experimental approaches including direct cloning, PCR, and/or Northern blotting [9], [26], were obtained from miRBase (Release13.0, March 2009; http://microrna.sanger.ac.uk). All of the above miRNAs were clustered by CD-HIT-EST [27] with c = 1, n = 8, d = 250, and g = 1. Only one mature sequence was selected from each cluster in order to eliminate closely related sequences; this approach generated 684 non-redundant mature miRNAs. All 62,344 *Poncirus trifoliata* ESTs were downloaded from the National Center for Biotechnology Information (NCBI) GenBank EST database (March 2009; http://www.ncbi.nlm.nih.gov/). All of these ESTs were screened against the 684 known miRNAs. Citrus unigenes were obtained from the C46 Database of HarvEST: Citrus 1.20, which displays 89 libraries and 229570 ESTs [14].

### Software availability

The comparative software (BLAST-2.2.14) was downloaded from NCBI GenBank. RNAfold was used to analyze the secondary structure of the RNAs. BLASTX from the web site http://www.ncbi.nlm.nih.gov/BLAST/ was used for the analysis of potential targets [28]. Putative citrus miRNAs were first blasted against the Harvest C46 Citrus unigene database on the Harvest Blast Search web server. BlastN hits with fewer than four nucleotides mismatches (plus/minus) were chosen as the candidate targets, which were then searched in Citrus Harvest 1.20 program using BlastX to obtain their putative functions [14].

### Prediction of potential miRNAs

The outline of our prediction procedure is according to a previously published method [6], [13]. First, we took one known miRNA from the reference set and aligned its seed region (positions 2–8) to the ESTs on both strands. At each hit position, a “raw miRNA” that had the same length as the known miRNA was extracted. Second, we took the known miRNA as a pattern and used PatScan [29] to filter all of the raw miRNAs, allowing a maximum of three mismatches between the known miRNA and the raw miRNAs. For the high conservation of mature sequences, particularly in the seed region [24], we stipulated that all of the mismatches could only occur in the non-seed region. The secondary structures of these sequences were predicted using RNAfold [30]. Finally, we explored a series of criteria to filter the sequences: (1) the predicted secondary structure had a high negative MFE; (2) the predicted mature miRNAs had no more than four nucleotide substitutions compared with other plant mature miRNAs; (3) the mature miRNA could be localized in one arm of the hairpin structure; (4) the RNA sequence could fold into an appropriate stem-loop hairpin secondary structure; (5) no loop or break in the miRNA or its opposite miRNA* sequences; (6) no more than 6 mismatches between the predicted mature miRNA sequence and miRNA* sequence in the secondary structure.

### Prediction of potential targets of miRNAs

Based on a transcriptome analysis in *Arabidopsis* transgenic plants over-expressing miRNAs, Schwab *et al.* [31] designed a set of rules for predicting miRNA targets. These criteria included the allowance for one mismatch in the region complementary to nucleotides 2 to 12 of the miRNA, but not at the cleavage site (nucleotides 10 and 11), and three additional mismatches between nucleotide positions 12 and 21, but no more than two continuous mismatches within this region. By adopting these rules to predict newly identified miRNA targets in citrus, we allowed one mismatch between the positions 1 to 9 from the 5′ end of the miRNA, no mismatches for positions 10 and 11, and another two mismatches between positions 12 and 21/24. The number of allowed mismatches at complementary sites between miRNA sequences and potential mRNA targets was no more than four, and no gaps were allowed at the complementary sites.

### Oligonucleotide synthesis and preparation

All the oligonucleotides used were purchased from Invetrogene Technologies, and then were purified by desalting. All primers used in this study are listed in Table 2.

### Low molecular RNA extraction

Roots, Leaves, young shoots, flowers, and fruits (1 cm diameter) were harvested from a seven-year-old trifoliate orange, and the total RNA was isolated from 100 mg of these tissues using TRIZOL reagent (Invitrogen, Life Technologies, Carlsbad, CA). Low molecular weight RNA and high molecular weight RNA were separated with 4M LiCl [14], [32]. The small RNA fraction was dissolved in 30 µl of RNase-free water. The concentration of the RNA was measured by the UV-1800 spectrophotometer (Shimadzu, Japan) and visually checked in a 2.0% agarose gel.

### Construction and screening of a cDNA library of small RNAs

We utilized the same procedure (Fig. 1A) to generate the miRNA-enriched library that has been popularly used to clone miRNAs and to measure the expression of miRNAs via RT-PCR [33]–[35], in which 5′- and 3′-end adaptors were linked to the miRNA molecules. Small RNAs were polyadenylated at 37°C for 60 min in a 50 µl reaction mixture with 1.5 µg of total RNA, 1 mM ATP, 2.5 mM MgCl_2_, and 4U poly(A) polymerase (Ambion, Austin, TX). Poly(A)-tailed small RNA was recovered by phenol/chloroform extraction and ethanol precipitation. A 5′ adapter (5′-CGACUGGAGCACGAGGACACUGACAUGGACUGAAGGAGUAGAAA-3′) was ligated to the poly(A)-tailed RNA using T4 RNA ligase (Invitrogen, Carlsbad, CA), and the ligation products were recovered by phenol/chloroform extraction followed by ethanol precipitation. Reverse transcription was performed using 1.5 µg of small RNA and 1 µg of (dT)_30_ RT primer (ATTCTAGAGGCCGAGGCGGCCGACATG-d(T)_30_ (A, G, or C) (A, G, C, or T)) with 200 U of SuperScript III reverse transcriptase (Invitrogen, Carlsbad, CA). Poly(A)-tailed small RNA (10 µl total volume) was incubated with 1 µl of (dT)_30_ RT primer and 1 µl dNTP mix (10 mM each) at 65°C for 5 min to remove any RNA secondary structure. The reactions were chilled on ice for at least 2 min, the remaining reagents [5×buffer, dithiothreitol (DTT), RNaseout, SuperScript III] were added as specified in the SuperScript III manual, and the reaction proceeded for 60 min at 50°C. Finally, the reverse transcriptase was inactivated by a 15 min incubation at 70°C. After the preparation of the miRNA libraries from various organs and tissues, we pooled similar quantities of these library samples for further PCR amplification reactions.

### Analyses of miRNA by 5′miR-RACE and 3′miR-RACE

The cDNA was amplified with the mirRacer 5′ primer (5′- GGACACTGACATGGACTGAAGGAGTA-3′) and the mirRacer 3′ primer (5′-ATTCTAGAGGCCGAGGCGGCCGACATG-3′) to generate a pool of non-gene-specific product. These miRacer primers are complementary to the 5′ and 3′ adaptors, respectively. The conditions used for the amplification were carried out for 25 cycles at a final annealing temperature of 60°C. 5′miR-RACE reactions were performed with the mirRacer 5′ primer and miRNA-gene-specific forward primers (GSP1), and 3′ miR-RACE reactions were carried out with the mirRacer 3′ primer and miRNA-gene-specific reverse primers (GSP2). GSP1 and GSP2 were complementary to 17 nucleotide length sequences of the potential ptr-miRNAs and a piece of Poly(T) and 5′ adaptor (Fig. 1B, Table 2). In each case, a unique gene-specific DNA fragment was amplified. After the amplification, the 5′ RACE and 3′ RACE PCR products were separated in a 2.5% agarose gel with ethidium bromide (EtBr) staining. The gel slices containing DNA with a size of about 60 bp (5′ RACE product) and 87 bp (3′ RACE product) were excised and the DNA fragments were purified using an agarose gel DNA purification kit (Takara, Japan), according to the manufacturer's instructions. The DNA fragment was directly sub-cloned with the TOPO TA cloning Kit (Invitrogen, USA). Colony PCR was performed using the PCR-specific primer pairs as above. The 5′ RACE and 3′ RACE clones with PCR products of about 60 bp and 87 bp, respectively, were sequenced. To check whether some mismatches between the sequence of the specific primer and that of the end sequence of the real miRNAs were allowed for workable PCR, we designed the primers of miR164 for miR-5′ RACE, miR-3′ RACE (Supplementary Table S1) that have 1–3 nucleotides being mismatched to the end sequence of the real sequence of miR164, the reaction using ptmir164 primers (GSP1, GSP2) were used as control.

### Real-Time PCR of miRNAs

The template for RT-PCR was the miRNA-enriched library mentioned above. To amplify the miRNA from the reverse transcribed cDNAs, we used the miRNA sequence as the forward primer (Table 2) and the mirRacer 3′Primer as the reverse primer. RT-PCR was conducted with the Rotor-Gene 3000 (Corbett Robotics, Australia) and the Rotor-Gene software version 6.1 [36]. For each reaction, 1 µL of diluted cDNA (equivalent to about 100 pg of total RNA) was mixed with 10 µL of 2× SYBR green reaction mix (SYBR® Green qRT-PCR Master Mix; Toyobo, Osaka, Japan), and 5 pmol each of the forward and the reverse primers were added in a final volume of 20 µL. The conditions for the PCR amplification were as follows: polymerase activation at 95°C for 1 min, then 95°C for 1 min, followed by 50 cycles of 95°C for 15 s, 95°C for 15 s, 60°C for 20 s, and 72°C for 20 s. The fluorescence signal was measured once every 1°C. Negative PCR controls (no cDNA template) were prepared to detect possible contamination. The specificity of the primer amplicons was checked by a melting curve analysis. The CT values were converted into relative copy numbers using a standard curve [37]. The 5.8S rRNA was previously used as a reference gene in the qPCR detection of miRNAs in *Arabidopsis* [38]. The data were analyzed with an R^2^ above 0.998 using the LinRegPCR program [39].

### Modified 5′ RNA ligase-mediated RACE for the mapping of mRNA cleavage sites

Total RNA was extracted from the leaf, stem, root, flower, and fruit tissues of an adult trifoliata orange tree using Trizol reagent. Poly(A)^+^ mRNA was purified from all kinds of pooled tissue RNA using the PolyA kit (Promega, Madison, WI), according to manufacturer's instructions. A modified procedure for 5′ RNA ligase-mediated RACE (RLM-5′RACE) was followed with the GeneRacer Kit (Invitrogen, CA), as described previously [14], [19]. The PCR amplifications were performed using the GeneRacer 5′ primer and the gene-specific primers (Table 4). Nested PCR amplifications were performed using the GeneRacer 5′ nested primer and the nested gene-specific nested primers (Table 4). The amplification products were gel purified, cloned, and sequenced, and at least 15 independent clones were sequenced.

**Table 4:** Primers used for modified 5′ RLM-RACE mapping of the miRNA cleavage sites and putative ptr-miRNA target genes.

miRNAs	Putative targets^A^	Target protein	Conserved gene in other plants (E-score)	Gene-specific primer	Nested gene-specific primer
ptrmir156	FJ502237	Pt-SPL9	AT2G42200 (3e-41)	TTAAAGGGACCAATGAATCTGCTGGTTGGAGT	ACAGGTTGAGCGACGGATTTGGTATGG
ptrmir156	FJ502238	Pt-SPL13	AT5G50670 (9e-49)	CTCCCAATGAAAGGGAATTGTTTGAG	GGTATCACTGGCTGCGGACCCATCAT
ptrmir164	FJ619349	Pt-NAC1	AT5G61430 (4e-103)	TCAATAATTCCAAAGACAATCAAGGGCTACT	AGGGCTACTGGGCCAGCAGAACTTG
ptr-mir167	UC46-16450	Pt-ARF8	AT5G37020 (3e-162)	ATGACGGTCACTTACTCCCATGGGTCGT	CAAGCAGTAGGAGACAAATCTTAACACAC
ptrmir171	GQ505957	Pt-SCL6	AT4G00150 (1e-37)	GCATAAGAGAAGCCCACTGCCCACCAT	ATCGGTGAGATTTCGGAGAAAGATTTGTAAGC
ptrmir319	GQ505958	Pt-TCP4	AT3G15030 (1e-59)	ATGGCGAGAATCAGAGGAAGCAGAGGA	CATCTTCGCCTTGGATTCGTGCTGG
ptrmir482a	UC46-36616	Pt-PRS5	AT4G10780 (1e-06)	GAGCTCCCGGGATCTCGAATCTTCTGAGTATACT	TCTTGGATTTTCTCCAAGTTGGCATC
ptrmir1446	GU072592	Pt-GRAS	IPR005202 (2e-141)	TTCGAAGGCGGCGTGGCGTTGGC	CGCGGTTGGCGAGGCTTTCGAT

A The target genes include two *P. trifoliate* orange unigenes (UC46-16450, UC46-36616) from the citrus EST database (Version 1.20 of HarvEST: Citrus, http://harvest.ucr.edu) and the deposited in NCBI.

## Discussion

To our knowledge, even though quite numbers of methods have been developed for computational prediction of miRNAs, the disadvantages of them in that the precise sequences of miRNAs usually cannot be determined and several candidate miRNA orthologs or paralogs might be predicted for a specific miRNA (e.g. the prediction of ptr-miR482 in this study) haven't been overcome experimentally. miR-RACE was the first experimental approach reported to overcome this problem, in which some challenging steps were employed.

From the amplification of the 5′ and 3′ ends of the miRNAs to be studied, the two gene-specific primers (GSP1, GSP2) were used to amplify their opposite ends well for their accurate end sequences. However, the ends complementary to the specific primers could not be amplified accurately due to the amplifications were determined by the primers designed. When the potential miRNAs predicted computationally were several nucleotides different from the true-to-type validated, the sequence corresponding to the specific primers were amplified exactly the same with those of the primers. This was due to the mismatches between primers and the true-to-type mature miRNAs, even though these mismatches were allowed for successful PCR amplification [40]. The specific primers covered 17 nucleotides of the candidate mature miRNAs was put forward both for relative high specificity of the primer to be designed to the corresponding miRNA and for meet of the criterion for the minimum number of nucleotides of a regular PCR primer. From our study and the identity level of plant miRNA orthologs, the number of 17 nucleotides of the miRNA chosen for primer design was workable and reasonable in determining the nucleotides flanking the last nucleotide in the predicted miRNA for this design would allow at most three and four mismatched nucleotides.

Even though all the ptr-miRNA sequence validation results were the same as those predicted computationally, it did not mean all the computational miRNA prediction methods can give the accurate sequences of potential miRNAs identified. The evolution of miRNA and parameters used in the prediction can influence the miRNA prediction. Sometimes, several potential miRNAs were predicted for a miRNA family [8]. We have also found the end sequence of some miRNAs predicted computationally in grapevine (*Vitis vinifera*) and apple (*Malus domestica*) were 1–3 nucleotides different from their corresponding true-to-type ones by this newly developed method (data not shown). The situation of the result that the termini nucleotides of ptr-miRNAs validated were the same as the predicted ones in this study can be explained as that the computational prediction method employed was powerful enough and can identify the true-to-type miRNAs at relative high efficiency, and not many ptr-miRNAs were predicted and validated. However, this does not influence the efficiency and workability of miR-RACE. With more miRNAs needed to be validated, some miRNAs that have some nucleotides different from their corresponding true-to-type could be verified.

## Supporting Information

Figure S1 The 5′ RACE and 3′ RACE products generated using primers (Table S1) with 1–3 nucleotide mismatched to ptr-miR164 were run in an ethidium bromide-stained agarose gel. The sizes of the molecular weight markers of the bottom and the second from bottom bands are 50 bp and 100bp, respectively. Lanes 1–4 are 5′RACE products from the PCR reactions in which primer ptr-miR164m3 (GSP1), ptr-miR164m2 (GSP1), ptr-miR164m1 (GSP1), and ptr-miR164 (GSP1) were used as one of the two primers needed, respectively, and lanes 5–8 are the 3′RACE products of primer ptr-miR164 (GSP2), ptr-miR164m1(GSP2), ptr-miR164m2 (GSP2), and ptr-miR164m3 (GSP2).
(0.20 MB TIF)

Table S1 The primers with nucleotides mismatched to ptmiR164 used in the verification of the workability of them in miR-RACE PCR amplifications and the partial sequences of the PCR products.
(0.03 MB DOC)
