# Supplementary figures and images for: MiR-RACE, a New Efficient Approach to Determine the Precise Sequences of Computationally Identified Trifoliate Orange (Poncirus trifoliata) MicroRNAs
Source: PLoS One. 2010 Jun 7;5(6):e10861. doi: 10.1371/journal.pone.0010861 (PMC2881865; doi:10.1371/journal.pone.0010861)

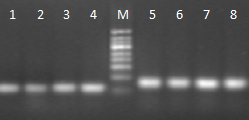

Supplement: Figure S1 — The 5′ RACE and 3′ RACE products generated using primers (Table S1) with 1–3 nucleotide mismatched to ptr-miR164 were run in an ethidium bromide-stained agarose gel. The sizes of the molecular weight markers of the bottom and the second from bottom bands are 50 bp and 100bp, respectively. Lanes 1–4 are 5′RACE products from the PCR reactions in which primer ptr-miR164m3 (GSP1), ptr-miR164m2 (GSP1), ptr-miR164m1 (GSP1), and ptr-miR164 (GSP1) were used as one of the two primers needed, respectively, and lanes 5–8 are the 3′RACE products of primer ptr-miR164 (GSP2), ptr-miR164m1(GSP2), ptr-miR164m2 (GSP2), and ptr-miR164m3 (GSP2). (0.20 MB TIF) [file pone.0010861.s001.tif]
