# Supplementary material for: MiR-RACE, a New Efficient Approach to Determine the Precise Sequences of Computationally Identified Trifoliate Orange (Poncirus trifoliata) MicroRNAs
Source: PLoS One. 2010 Jun 7;5(6):e10861. doi: 10.1371/journal.pone.0010861 (PMC2881865; doi:10.1371/journal.pone.0010861)
Supplement: Table S1 — The primers with nucleotides mismatched to ptmiR164 used in the verification of the workability of them in miR-RACE PCR amplifications and the partial sequences of the PCR products. (0.03 MB DOC) [file pone.0010861.s002.doc]

**Table S1 The primers with nucleotides mismatched to ptmiR164 used in the verification of the workability of them in miR-RACE PCR amplifications and the partial sequences of the PCR products.**

| Primer | Sequence (5'-3') | Product size (bp) | Partial sequence of miR-RACE product (5'-3') |
| --- | --- | --- | --- |
| ptr-mir164 (GSP1) | TTTTTTTTTTGCACGTGCCCTGCTTCT | 61bp | TTTTTTTTTT**TGCACGTGCCCTGCTTCTCCA*** |
| ptr-mir164 (GSP2) | GGAGTAGAAATGGAGAAGCAGGGCACG | 87bp | GGAGTAGAAA**TGGAGAAGCAGGGCACGTGCA** |
| ptr-mir164m1 (GSP1) | TTTTTTTTTTGCACGTGCCATGCTTCT | 61bp | TTTTTTTTTT**TGCACGTGCCATGCTTCTCCA*** |
| ptr-mir164m1 (GSP2) | GGAGTAGAAATGGAGAAGCAGGGCATG | 87bp | GGAGTAGAAA**TGGAGAAGCAGGGCATGTGCA** |
| ptr-mir164m2 (GSP1) | TTTTTTTTTTGCACGTGCCCTGCGACT | 61bp | TTTTTTTTTT**TGCACGTGCCCTGCGACTCCA*** |
| ptr-mir164m2 (GSP2) | GGAGTAGAAATGGAGAAGGAGGGCTCG | 87bp | GGAGTAGAAA**TGGAGAAGGAGGGCTCGTGCA** |
| ptr-mir164m3 (GSP1) | TTTTTTTTTTGCACGTGGGGTGCTTCT | 61bp | TTTTTTTTTT**TGCACGTGGGGTGCTTCTCCA*** |
| ptr-mir164m3 (GSP2) | GGAGTAGAAATGGAGTTGCAGGGCAAG | 87bp | GGAGTAGAAA**TGGAGTTGCAGGGCAAGTGCA** |

Underlined bases are of the primers for miR-5’Race and miR-3’Race, respectively; Base sequences in bold represent the ptr-miR164 region in the sequenced products of miR-5’RACE and miR-3’RACE; Red bases represent the randomly artificial ones mismatched to ptr-miR164; * point reverse complementary sequence of ptr-miR164; Bases in bold non-underlined are those at ends of ptr-miR164 validated.
